# Can living donor liver transplantation offer similar outcomes to deceased donor liver transplantation using expanded selection criteria for hepatocellular carcinoma?

**DOI:** 10.12669/pjms.314.7523

**Published:** 2015

**Authors:** Li-Ping Chen, Chuan Li, Tian-Fu Wen, Lu-Nan Yan, Bo Li, Jia-Yin Yang

**Affiliations:** 1 Li-Ping Chen, Department of Biliary Surgery, West China Hospital of Sichuan University, Chengdu-610041, China. Department of Liver Surgery & Liver Transplantation, West China Hospital of Sichuan University, Chengdu-610041, China; 2 Chuan Li, Department of Liver Surgery & Liver Transplantation, West China Hospital of Sichuan University, Chengdu-610041, China; 3 Tian-Fu Wen, Department of Liver Surgery & Liver Transplantation, West China Hospital of Sichuan University, Chengdu-610041, China; 4 Lu-Nan Yan, Department of Liver Surgery & Liver Transplantation, West China Hospital of Sichuan University, Chengdu-610041, China; 5 Bo Li, Department of Liver Surgery & Liver Transplantation, West China Hospital of Sichuan University, Chengdu-610041, China; 6 Jia-Yin Yang, Department of Liver Surgery & Liver Transplantation, West China Hospital of Sichuan University, Chengdu-610041, China

**Keywords:** Living donor liver transplantation, Deceased donor liver transplantation, Hepatocellular carcinoma, Milan criteria, Outcomes, Alpha-fetoprotein (AFP), Deceased donor liver transplantation (DDLT), Hepatocellular carcinoma (HCC), Living donor liver transplantation (LDLT), Model for end-stage liver disease (MELD), Microvascular invasion (MVI), Recurrence-free survival (RFS) rate and overall survival (OS), University of California, San Francisco (UCSF)

## Abstract

**Objective:**

To compare the outcomes of living donor liver transplantation (LDLT) versus deceased donor liver transplantation (DDLT) for patients with hepatocellular carcinoma (HCC) in different selection criteria.

**Methods:**

Data of patients with HCC who underwent liver transplantation between 2005 and 2013 at our center were reviewed. Clinical data of LDLT recipients and DDLT recipients were compared. The postoperative recurrence-free survival (RFS) rate and overall survival (OS) rate after LDLT versus DDLT were compared in the Milan recipients, the University of California, San Francisco (UCSF) recipients, the up-to-seven recipients, the Hangzhou recipients and the Chengdu recipients.

**Results:**

Data of 255 patients were retrospectively reviewed in this study. Seventeen DDLT recipient and 9 LDLT recipients died during the perioperative period. Among the remaining 229 recipients (N_LDLT_=66, N_DDLT_=163), 96 patients met the Milan criteria, 123 recipients met the UCSF criteria, 135 patients met the up-to-seven criteria, 216 patients met the Hangzhou criteria, and 229 recipients met the Chengdu criteria. The overall RFS and OS rates of the Milan recipients, the UCSF recipients, the up-to-seven recipients, the Hangzhou recipients and the Chengdu recipients after LDLT and DDLT were all similar.

**Conclusion:**

Using well-studied selection criteria, LDLT offers similar outcomes to DDLT for patient with HCC, even using expanded selection criteria.

## INTRODUCTION

Hepatocellular carcinoma (HCC) is the sixth most common malignancies and third most frequent cancer related death in the world.^1^ Liver transplantation is widely accepted as a curative treatment for patients with HCC. Liver transplantation can removal whole tumors and cure the background liver disease. However, worldwide scarcity of deceased donor liver grafts remains to be a great limitation of this management. About 20%-30% patients dropped from the waiting list due to tumor progression during the extended period of waiting for liver transplantation.^1^ Living donor liver transplantation (LDLT) is perceived as an alternative life-saving treatment to deceased donor liver transplantation (DDLT). Theoretically, LDLT can expand the donor pool. The use of LDLT may shorten waiting time and possibility decrease the mortality of waiting list. However, the outcomes of LDLT for HCC remain controversial.^2^,^3^ Some clinical investigations have reported the long-term survival rates of LDLT versus DDLT may be not different, but the incidence of postoperative recurrence was higher following LDLT.^2^ Another investigator argued the outcomes of LDLT for HCC were comparable to DDLT.^3^ Conclusions of meta-analyses regarding this issue also showed opposite views Recently, a meta-analysis performed by Liang et al.^4^ suggested the recurrence-free and long-term survival rates following LDLT were equal to DDLT. However, another meta-analysis performed by Grant et al.^5^ around the same period showed LDLT had a lower recurrence-free survival rate than DDLT.

Milan criteria (one nodule with a maximal diameter of 5 centimeters; or up to 3 nodules with a maximal diameter of 3 centimeters; without vascular invasion) are generally accepted as the golden standard to select patients with HCC of liver transplantation.^6^ Patients with HCC within Milan criteria have a comparable survival rates to individuals transplanted for benign liver diseases. However, the Milan criteria are too stringent and may deny HCC patients who may benefit from liver transplantation. Subsequently, a number of investigations attempted to expand Milan criteria. In 2001, Yao et al.^7^ confirmed appropriate expansion of the Milan criteria did not negatively impact HCC patient survival and proposed the University of California, San Francisco (UCSF) criteria (single tumour up to 6.5cm in maximum diameter; or up to three tumours with none larger than 4.5cm and with a total tumour diameter no more than 8 cm). In 2008, Zheng et al.^8^ introduced the Hangzhou criteria^8^, which, without the presence of macrovascular invasion and exhepatic metastasis, must fulfill one of the two following requirements: (a) total tumor diameter less than or equal to 8 cm or (b) total tumor diameter greater than 8 cm, well or moderate differentiation and a preoperative alpha-fetoprotein (AFP) level of no more than 400 ng/mL. In 2009, Mazzaferro and colleague even proposed up-to-seven criteria (tumor number and maximal tumor size no greater than 7) to expand Milan criteria.^9^ In our center, we used Chengdu criteria^10^ (total tumor size up to 9 cm regardless of tumor number and without macrovascular invasion and exhepatic metastasis) to select HCC patients. There were some well-studied selection criteria for LDLT in Asia, such as Tokyo criteria^11^ (up to 5 tumours with a maximum diameter of 5 cm). Most published investigations with respect to comparing outcomes of LDLT versus DDLT for HCC used Milan criteria.^12^ However, a number of transplant centers selected HCC candidates of liver transplantation using expanded selection criteria.^8^,^10^ Whether LDLT could achieve similar outcomes to DDLT when using expanded selection criteria was not established. In the present study, we attempted to compare the outcomes of patients with HCC underwent LDLT versus DDLT using different selection criteria.

## METHODS

### Study group

Between 2005 and 2013, 255 patients underwent liver transplantation at our center had a diagnosis of HCC within Chengdu criteria. HCC was confirmed by pathological examination of the explanted liver. In different transplant criteria, patients were divided into two groups: LDLT group and DDLT group. Clinical data of recipients, including tumor size, tumor number, differentiation, microvascular invasion (MVI), alpha-fetoprotein (AFP), age, model for end-stage liver disease (MELD) score, and so on were reviewed. MELD score was calculated using the following formula: MELD score =9.57×Ln creatinine (mg/dL)+11.2×6(Ln INR)+3.78×Ln bilirubin (mg/dL)+6.43.^13^ According to the difference of transplant graft, patients were divided into LDLT group and DDLT group. Outcomes of HCC after LDLT versus DDLT were compared in the Milan recipients, the UCSF recipients, the up-to-seven recipients, the Hangzhou recipients and the Chengdu recipients. This study and all transplantations were approved by the ethics committee of West China Hospital.

### Donor selection

Donors must be the ABO blood type compatible and have negative laboratory findings. For LDLT, donors must be close relatives. Volumetric computed tomography with contrast was administrated to assess the liver of all donors. The right hepatic lobe of donors without middle hepatic vein must be at least 0.8% of the recipient’s standard weight and the remaining liver remnant in the donor must be at least 40%.

### Follow-up

After transplantation, recipients were regularly monitored by serum AFP examination, visceral ultrasonography or CT or MR imaging and chest radiography every 3 months. Bone scintigraphy was performed whenever HCC recurrence was suspected. Recurrence was defined as positive imaging findings compared to preoperative examination values and newly rising tumor marker (AFP) values or confirmed by biopsy or resection.^14^

### Immunosuppression and antivirus protocols

After liver transplantation, immunosuppression consisted of tacrolimus or cyclosporine, mycophenolate mofetil and steroid. Steroid pulse therapy was conducted in patients with rejection. Steroid was tailed off as early as possible. Lamivudine and hepatitis B immune globulin was administered to prevent hepatitis B virus recurrence for HBsAg positive patients after transplantation. Moreover, hepatitis B immune globulin was given to hepatitis B virus patients during transplantation.

### Statistical analysis

All continuous variables are presented as the mean±SD and compared using one-way analysis of variance. A chi-square test or Fisher’s exact test was performed for categorical variables. The Kaplan-Meier method with log-rank test was performed to compare the recurrence-free and long-term survival of different groups. All statistical analyses were performed using SPSS 21.0 for Windows. A *p* value less than 0.05 was considered significant.

## RESULTS

**Fig. 1:**
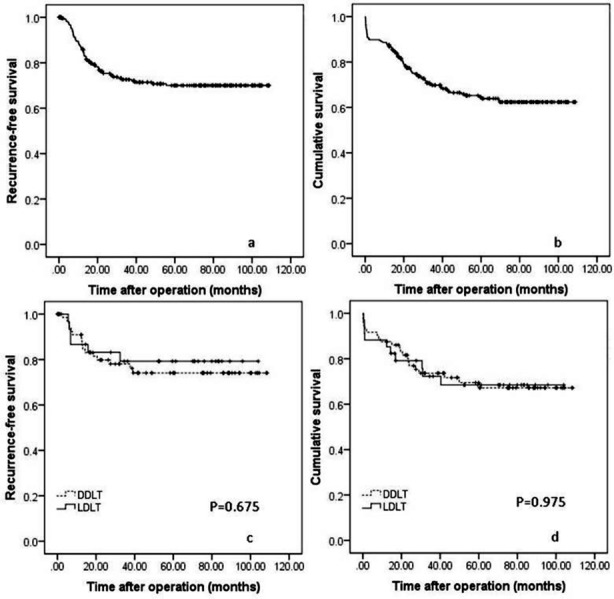
RFS (Fig.1a) and OS (Fig.1b) curves of all patients. Comparison of the RFS (Fig.1c) and OS (fig.1d) rates of LDLT and DDLT for patients with HCC within Milan criteria.

A total of 255 patients, including 180 DDLT recipients and 75 LDLT reciients, underwent liver transplantation at our center from 2005 to 2013. 26 (10.20%) patients, including 17 (9.44%) DDLT recipients and 9 (12%) LDLT recipients, died during the perioperative period. The causes of death included multiple organ dysfunction syndrome (N=14), infections (N=7), renal failure (N=3) and bleeding (N=2). The perioperative mortality rate of LDLT was slightly higher than DDLT. However, this difference didn’t reach statistically significant (*P*=0.539). Recurrences and recurrence-related deaths of the remaining 229 recipients, including 163 DDLT recipients and 66 LDLT recipients, were analyzed. The mean age of recipients was 47.32±9.07 years, whereas the mean donor age was 34.23±8.59 years. The mean MELD score was 11.77±5.95. The mean tumor size was 5.23±2.26 cm. MVI was observed in 77 patients. One hundred three patients had an AFP level more than 400ng/ml. The mean waiting time of DDLT recipients was 46.88±32.12 days, which was significantly longer than LDLT patients (23.37±16.32 days, *P*<0.001). In the present study, 106 patients met the Milan criteria, 137 recipients met the UCSF criteria, 109 recipients met the Tokyo criteria, 152 patients met the up-to-seven criteria, 242 patients met the Hangzhou criteria, and all patients (N=255) met the Chengdu criteria. The mean follow-up time was 52.45±31.31 months. During the follow-up period, 62 recipients suffered from post-transplant recurrence. A total of 83 recipients died during the follow-up period. The 1-, 3-, 5-year recurrence-free survival of all recipients were 86.0%, 72.6% and 70.0% (Fig.1a), whereas the 1-, 3-, 5-year overall survival rates of all recipients were 87.8%, 69.8% and 64.6% (Fig.1b).

### Outcomes of LDLT versus DDLT for HCC patients within Milan criteria

In the present study, 106 patients with HCC met the Milan criteria, including 34 LDLT recipients and 72 DDLT recipients. The 1-, 3-, 5-year recurrence-free survival rates of HCC patients within Milan criteria were 90.9%, 78.0%, and 74.1% for DDLT versus 86.7%, 79.2% and 79.2% for LDLT respectively, with no significant difference was observed (Fig.1c, *P*=0.675). The overall patient survival rates at 1, 3 and 5 years after liver transplantation were 88.2%, 73.7% and 69.5% respectively after DDLT, and 88.2%, 72.3% and 68.5% respectively after LDLT (Fig.1d, *P*=0.975).

### Outcomes of LDLT versus DDLT for HCC patients within UCSF criteria

**Fig. 2:**
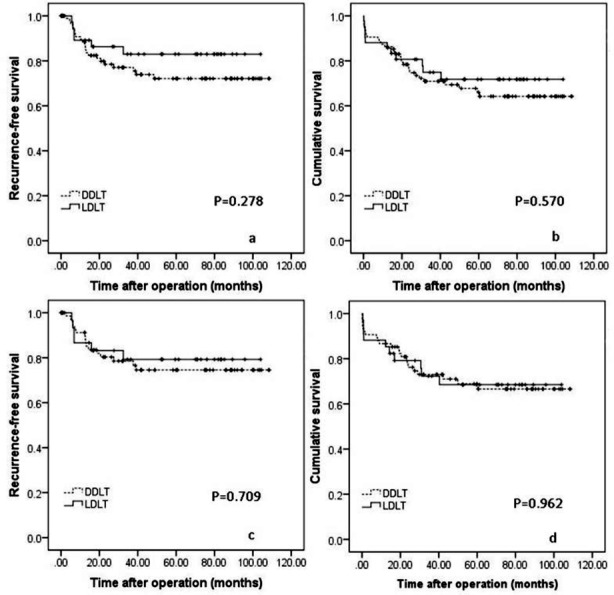
Comparison of the RFS (figure 2a) and OS (figure 2b) rates of LDLT and DDLT for patients with HCC within UCSF criteria. RFS (figure 2c) and OS (figure 2d) curves of LDLT versus DDLT for patients with HCC within up-to-seven criteria

When the University of California San Francisco criteria was applied, 137 recipients, including 95 DDLT recipients and 42 LDLT recipients, fulfilled the selection criteria. The 1-, 3-, 5-year RFS of LDLT recipients were 89.2%, 83.0% and 83.0% respectively, which were slightly higher than the DDLT recipients (88.4%, 77.1% and 72.1% for 1-, 3-, 5-year RFS rates respectively). However, this difference didn’t reach statistical significance (Fig.2a, *P*=0.278). The 1-, 3-, 5-year OS rates of DDLT recipients were 86.3%, 70.9% and 66.0% respectively, whereas the 1-, 3-, 5-year OS rates of LDLT recipients were 88.1%, 74.9% and 71.8% respectively (Fig.2b, *P*=0.570).

### Outcomes of LDLT versus DDLT for HCC patients within Tokyo criteria

There were 109 patients meeting Tokyo criteria, including 75 DDLT recipients and 34 LDLT recipients. The 1-, 3-, 5-year RFS of LDLT and DDLT recipients were 86.7%, 79.2%, 79.2% and 91.2%, 78.5%, 74.5%respectively (Fig.2c, *P*=0.709). The 1-, 3-, 5-year OS rates of DDLT recipients were 86.7%, 73.0% and 68.9% respectively, whereas the 1-, 3-, 5-year OS rates of LDLT recipients were 88.2%, 72.3% and 68.5% respectively (Fig.2d, *P*=0.962).

### Outcomes of LDLT versus DDLT for HCC patients within up-to-seven criteria

**Fig. 3:**
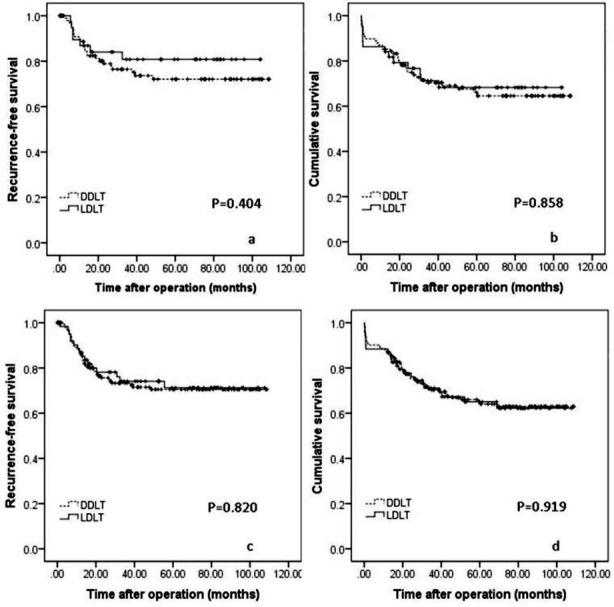
Comparison of the RFS (Fig.3a) and OS (Fig.3b) rates of LDLT and DDLT for patients with HCC within Hangzhou criteria. RFS (Fig.3c) and OS (Fig.3d) curves of LDLT versus DDLT for patients with HCC within Chengdu criteria.

In this study, 152 recipients, including 44 LDLT recipients and 108 DDLT recipients, met the up-to-seven criteria. The 1-, 3-, 5-year RFS of LDLT recipients were 86.8%, 80.8% and 80.8% respectively, which was similar to DDLT recipients (88.7%, 76.4% and 72.1%; Fig.3a, *P*=0.404). The 1-, 3-, 5-year OS of two groups were also comparable (86.4%, 71.2% and 68.3% for LDLT recipients versus 85.2%, 70.4% and 66.1% for DDLT recipients respectively; Fig.3b, *P*=0.858).

### Outcomes of LDLT versus DDLT for HCC patients within Hangzhou criteria

When the Hangzhou criteria was applied, 242 recipients, including 173 DDLT recipients and 69 LDLT recipients met the selection criteria. The RFS rates at 1, 3, 5 years were 86.9%, 74.1% and 71.3% respectively for LDLT recipients, and 86.5%, 73.3% and 70.5% respectively for DDLT recipients (Fig.3c, *P*=0.820). The 1-, 3-, 5-year OS rates were 87.0%, 71.1% and 65.1% for LDLT recipients, and 87.3%, 70.3% and 65.0% for DDLT recipients (Fig.3d, *P*=0.919).

### Outcomes of LDLT versus DDLT for HCC patients within Chengdu criteria

**Fig. 4:**
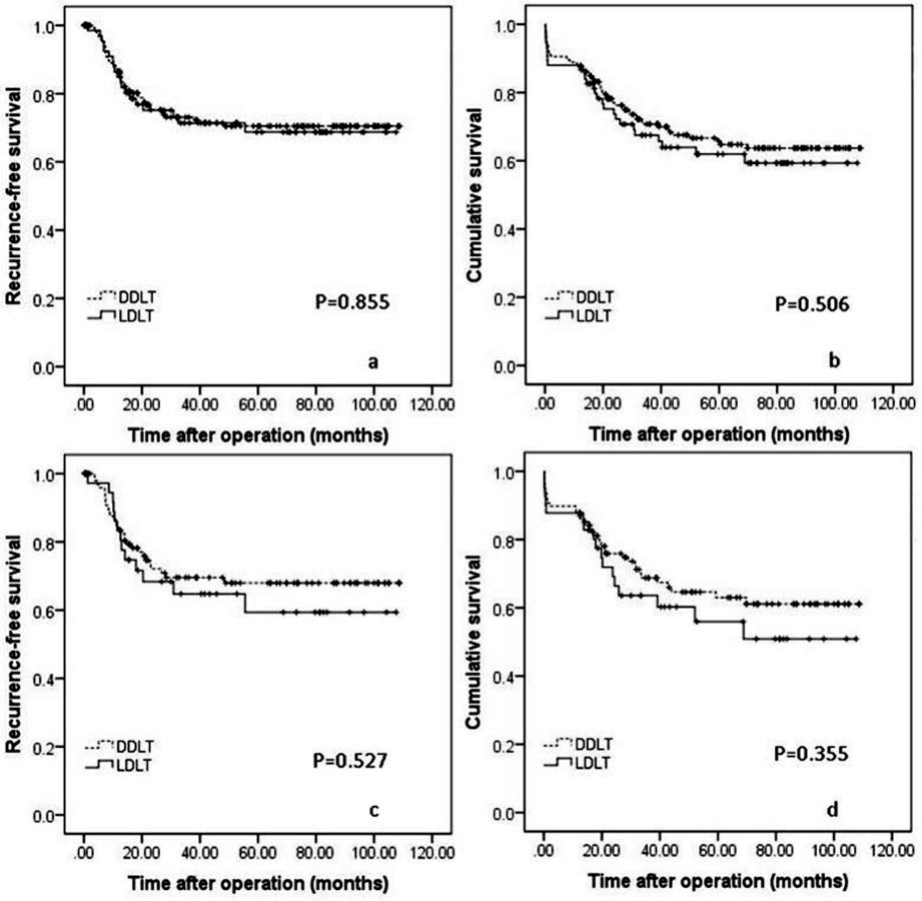
RFS (Fig.4a) and OS (Fig.4b) for patients outside of Milan criteria following LDLT versus DDLT. RFS (Fig.4c) and OS (Fig.4d) curves for patients in the Milan, UCSF, up-to-seven, Hangzhou and Chengdu criteria following liver transplantation.

In the current study, all patients (N=255) fulfilled the Chengdu criteria. The 1-, 3-, 5-year RFS rates were 84.8%, 71.4% and 68.7% respectively for LDLT recipients (N=75), and 86.5%, 73.1% and 70.5% respectively for DDLT recipients (N=180). No significant difference was observed (Fig.4a, *P*=0.855). The 1-, 3-, 5-year OS rates of two groups were also similar (88.0%, 67.5% and 61.9% for LDLT recipients versus 87.8%, 70.8% and 65.7% for DDLT recipients; Fig.4b, *P*=0.506).

### Outcomes of patients outside of Milan criteria following liver transplantation

A total of 133 patients, including 97 DDLT recipients and 36 LDLT recipients, beyond Milan criteria in the present study. The 1-, 3- 5-year RFS rates were 83.3%, 64.7% and 59.4% for LDLT recipients versus 83.5%, 69.6% and 68.0% for DDLT recipients respectively (Fig.4c, *P*=0.527). The 1-, 3- 5-year OS for LDLT and DDLT recipients were 87.8%, 63.6% and 56.0% versus 88.0%, 68.8% and 63.0% respectively (Fig.4d, *P*=0.355).

## DISCUSSION

Liver transplantation was perceived as a curative treatment for patients with HCC. However, the gap between organ availability and transplant demand continues to widen. LDLT is perceived to be an effective choice to expand the donor pool and has saved many lives in the past two decades. However, the outcomes of LDLT for patients with HCC remains controversial. In the present study, we confirmed LDLT can offer a similar RFS and OS to DDLT, even in expanded selection criteria.

A number of studies suggested LDLT had a worst outcomes than DDLT because of the short waiting time and the surgical procedure of LDLT.^2^ The waiting time of DDLT was longer than LDLT, which was also confirmed by our study. Some patients with HCC may drop from the waiting list owing to tumor progression.^1^ In other words, long waiting time may be a method to assess the biological behavior of the tumor. Patients with high-grade malignant tumor may be apt to suffer from tumor progression during waiting for liver transplantation. LDLT was called “fast-track” transplantation. Patients with HCC may receive LDLT in a short waiting time. Accordingly, some patients with high-grade malignant tumor may be involved in the LDLT group. In addition, LDLT needs to preserve greater length of hepatic artery, portal vein, bile duct and the native vena cava. All of these may result in tumor remnants which can lead to postoperative recurrence. Moreover, LDLT transplanted a partial liver to the recipient. The graft size of LDLT is smaller than DDLT. Previous basic studies and clinical experience have demonstrated that hepatic sinusoid of small-for-size graft may be damaged by the excessive portal venous flow and the transient portal hypertension.^15^ The severe shear stress from the portal hemodynamic force can trigger a series of inflammatory reaction which provide a favorable environment for tumor recurrence.^16^ Additionally, the regeneration of the small-for-size graft following liver transplantation is also an angiogenesis-associated phenomenon.^17^ Furthermore, LDLT has more manipulation during liver transplantation, tumor cells may disseminate to other sites via the hepatic vein. All of the above-mentioned surgical procedures may contribute to recurrence after liver transplantation. Although LDLT has above-mentioned risk factors of postoperative recurrence, our study showed the postoperative RFS and OS rates of LDLT were comparable to DDLT. Moreover, our study confirmed LDLT could achieve similar outcomes to DDLT even in different expanded selection criteria. A number of previous investigations have confirmed tumor size, poor differentiation, presence of MVI and/or macrovascular invasion and a high level of preoperative AFP contributed to postoperative recurrence.^8^ Just as Table-I shows these risk factors were similar between the LDLT group and DDLT group in the present study. Our study also confirmed the RFS and OS of patients outside of Milan criteria also had a similar outcomes following LDLT versus DDLT.

**Table-I:** Demographic data of LDLT and DDLT recipients.

Variables	DDLT	LDLT	*P*
Donor variables		
Age (years)	34.14±7.36	34.44±11.13	0.812
Female/male	8/155	22/44	<0.001
Body mass index	22.79±2.95	22.54±11.13	0.531
Recipient variables		
Age (years)	47.93±9.51	45.82±7.72	0.110
Female/male	19/144	6/60	0.573
Body mass index	22.26±4.56	22.50±5.32	0.735
Creatinine (μmol/L)	77.89±27.99	73.25±18.76	0.217
Total bilirubin (μmol/L)	52.89±90.26	36.23±8.23	0.123
Albumin (g/L)	37.74±7.09	36.23±8.23	0.166
International normalized ratio	1.18±0.52	1.18±0.32	0.999
AFP > 400 ng/mL	72/163	31/66	0.700
Tumor size (cm)	5.24±2.24	5.22±2.31	0.949
MVI	50/163	27/66	0.138
Differentiation			0.694
Well	27	13	
Moderate	110	45	
Poor	26	8	
MELD score	12.03±6.44	11.12±4.50	0.296

Since the Milan criteria was proposed by Mazzaferro et al.^6^ in 1996, a number of models have been developed to expand the indications for liver transplantation for patients with HCC without compromising patient’s postoperative outcomes.^7^,^8^ In 2009, even Mazzaferro and colleague proposed up-to-seven criteria to expand Milan criteria.^9^ In China, there is lack of a national selection criteria of patients with HCC for liver transplantation. In the present study, only 96 (41.91%) patients fulfilled the Milan criteria. Although more than half of patients beyond the Milan criteria, current study confirmed UCSF criteria, up-to-seven criteria, Hangzhou criteria and Chengdu criteria let more patients have a chance to receive liver transplantation without increasing the risk of HCC recurrence after liver transplantation. We acknowledge, except the above-mentioned five selection criteria, there are many good selection criteria which were used in other transplant centers, such as the Asan criteria and Tokyo criteria.^18^ Milan criteria, UCSF criteria and up-to-seven criteria were most used selection criteria for HCC liver transplantation in the worldwide, whereas Hangzhou criteria and Chengdu criteria were two most common used criteria in liver transplantation for HCC in China. Accordingly, in the present study, we compared the outcomes of LDLT versus DDLT for patients with HCC within these five selection criteria.

The incidence of peri operative mortality after liver transplantation was 10.12% in our study, which was slightly higher than some reports^19^, but also comparable to a number of previous investigations.^20^ In 2005, our liver transplantation program, especially the LDLT program, hadjust started in a short period. Our previous study have confirmed learning curve played a key role in decreasing postoperative incidence of complications and mortality.^21^ So, this may be why our perioperative mortality was different than some studies. Moreover, LDLT has a smaller biliary and vascular calibre and an additional transection step, which may potentially increase the surgical risk and the incidence of postoperative complications.^22^ However, our study confirmed the incidence of perioperative mortality after LDLT was equal to DDLT. LDLT also has some advantages over DDLT: shorter cold and warm ischemic times, and a better organisation of the surgery time.^23^ These advantages may also contribute to a good long-term survival following LDLT.

### Limitations of the study

This is a single center analysis with a small sample size of LDLT group. We believe a larger series and a multicenter study design would minimize these limitations. Moreover, LDLT group had more female donors in this study. Just as previous investigations have reported tumor characteristics, such as poor differentiation, presence of macrovascular invasion, were the predominant factors affecting postoperative recurrence.^8^,^24^ Some investigations suggested factors associated with poor long-term survival following liver transplantation were high MELD score, older donor, presence of pre transplant renal failure, longer ischemic time and so on.^25^ Accordingly, we suggested that the difference of donor gender between two groups had little influences on the final conclusion. In conclusion, our study suggested LDLT can offer a similar outcomes to DDLT for patients with HCC when using expanded selection criteria.
